# The Initial Experience of Balloon Pulmonary Angioplasty for Chronic Thromboembolic Pulmonary Hypertension in Latvia

**DOI:** 10.3390/medicina60040568

**Published:** 2024-03-30

**Authors:** Anna Krigere, Verners Roberts Kalejs, Ricards Kaulins, Ainars Rudzitis, Liga Bondare, Matiss Sablinskis, Aivars Lejnieks, Dana Kigitovica, Marcin Kurzyna, Andris Skride

**Affiliations:** 1 Department of Rare Diseases, Pauls Stradins Clinical University Hospital, 1002 Riga, Latvia; anna.krigere@stradini.lv (A.K.); v.r.kalejs@gmail.com (V.R.K.); ainars_rudzitis@hotmail.com (A.R.);; 2 Department of Internal Diseases, Riga Stradins University, 1007 Riga, Latvia; kaulinsricards@gmail.com (R.K.); aivars.lejnieks@rsu.lv (A.L.); 3 Department of Internal Diseases, Riga East Clinical University Hospital, 1038 Riga, Latvia; 4 Department of Pulmonary Circulation, Thromboembolic Diseases and Cardiology, Center of Postgraduate Medical Education, ERN-LUNG Member, 05-400 Otwock, Poland

**Keywords:** balloon pulmonary angioplasty, chronic thromboembolic pulmonary hypertension, Latvia

## Abstract

*Background*: Treatment options for inoperable chronic thromboembolic pulmonary hypertension (CTEPH) or persistent pulmonary hypertension after pulmonary endarterectomy (PEA) include targeted medical therapy and balloon pulmonary angioplasty (BPA). BPA is an emerging treatment modality that has been reported to improve functional capacity, pulmonary hemodynamics, and right ventricular function. Reports from expert centers are promising, but more data are needed to make the results more generalizable. *Materials and Methods*: We conducted a prospective analysis of nine consecutive CTEPH patients who underwent balloon pulmonary angioplasty (BPA) sessions at Pauls Stradins Clinical University Hospital in Riga, Latvia between 1 April 2022 and 1 July 2023. We assessed World Health Organization (WHO) functional class, 6 min walk distance (6MWD), blood oxygen saturation (SpO_2_), brain natriuretic peptide (BNP) level at baseline and 3 months after the first BPA session. For two patients on whom repeated BPA sessions were performed, we additionally assessed cardiac output (CO), pulmonary vascular resistance (PVR), and mean pulmonary artery pressure (mPAP). *Results*: A total of 12 BPA procedures for nine patients were performed; repeated BPA sessions were performed for two patients. Our results show a reduction in BNP levels and improvement in WHO functional class, 6MWD, and SpO_2_ after the first BPA session. Improvement in 6MWD was statistically significant. Additionally, an improvement in pulmonary hemodynamic parameters was observed. *Conclusions*: Our data show that BPA is an effective interventional treatment modality, improving both the pulmonary hemodynamics and functional status. Moreover, BPA is safe and excellently tolerated.

## 1. Introduction

Chronic thromboembolic pulmonary hypertension (CTEPH) arises when a pulmonary embolism (PE) fails to resolve, leading to the accumulation of fibrous tissue that obstructs pulmonary arteries. CTEPH is characterized pathophysiologically by organized thromboembolic material, and by altered vascular remodeling initiated or potentiated by a combination of defective angiogenesis, impaired fibrinolysis, and endothelial dysfunction [1]. The changes in pulmonary vasculature lead to pulmonary hypertension (PH), which is defined as mean pulmonary artery pressure (mPAP) > 20 mmHg and pre-capillary PH combination of mPAP > 20 mmHg, pulmonary arterial wedge pressure (PAWP) ≤ 15 mmHg, and pulmonary vascular resistance (PVR) > 2 Wood units [2].

The reported incidence of CTEPH ranges from 0.9 to 5.7 per million, with a prevalence of 8 to 40 per million [2,3]. However, these figures are likely to be underestimated due to under-reporting [2,3]. In Latvia, the calculated incidence of CTEPH in 2016 was 5.1 per million population, with a prevalence of 15.7 per million population, placing it among the highest in Europe [4]. It is estimated that 0.56% to 3% of acute PE cases progress to CTEPH [5]. The resulting pulmonary hypertension, along with mechanical blockages and secondary vascular disease, causes right ventricular (RV) remodeling, dilation, exercise intolerance, RV failure, and premature mortality [6]. Patients who do not undergo surgery have a 70% survival rate after 3 years [6]. While drug therapy has a limited effect, preliminary data suggest that riociguat, a soluble guanylate cyclase (sGC) inhibitor, may improve medium-term survival [7].

Pulmonary endarterectomy (PEA) is recognized as the preferred treatment for those with CTEPH [8,9,10]. Yet, this significant surgical intervention comes with a roughly 2.2% risk of intraoperative mortality and nearly half of the patients face complications, even when performed in expert center. As a result, in some patients the potential risks are more substantial than the anticipated benefits [11]. Furthermore, when thromboembolic changes are located in the distal vasculature, PEA might not be an option. This leads to about 40% of patients being considered unsuitable for surgery, and out of those who do undergo PEA, around 20% might not achieve full clearance, leading to functional restrictions [12]. Patients with inoperable or residual CTEPH have the option of pulmonary hypertension-targeted drug therapy, which has demonstrated short-to-medium-term hemodynamic and symptomatic improvement [13,14,15,16] or balloon pulmonary angioplasty (BPA), which has only been supported by observational data thus far [17,18].

BPA has emerged as a novel technology that initially received limited attention due to its association with severe and potentially life-threatening complications such as reperfusion edema, pulmonary hemorrhage, and RV failure. However, over the past 15 years, BPA has regained prominence as centers in Japan and Europe have refined the interventional approach. They have adopted advanced imaging technology, employed a cautious staged procedure, and utilized smaller balloons to minimize damage to the pulmonary vessels [19]. A recent meta-analysis of a total of 1763 patients studied shows that BPA has become a safe and effective treatment option, with significant improvements in hemodynamic parameters, improved exercise tolerance and a relatively low risk of major complications [20]. The technique involves the use of catheters equipped with balloons, guided by wires, to address blockages in segmental and subsegmental pulmonary arteries [19]. BPA has shown compelling evidence of improving symptoms and hemodynamics by restoring blood flow [21]. A meta-analysis suggests that BPA may outperform drug therapy and potentially rival the efficacy of PEA [17]. However, no randomized control data comparing treatment strategies have been published to date [17]. Encouragingly, the benefits of BPA seem to endure for up to 5 years, with a low incidence of restenosis [22,23].

The latest guidelines from the European Society of Cardiology on pulmonary hypertension now give BPA a Class I recommendation for cases of inoperable and persistent chronic thromboembolic pulmonary hypertension [2].

Although long-term outcomes following BPA remain under-researched, and PEA typically offers greater reduction in pulmonary pressures, both treatments show comparably favorable effects on exercise capacity improvement [24].

In smaller countries such as Latvia, where access to pulmonary endarterectomy—the gold standard treatment for chronic thromboembolic pulmonary hypertension—may be nonexistent, and can only be treated abroad, the introduction of alternatives such as BPA represents a significant advancement in patient care.

Our approach to introducing BPA in our country has marked a significant leap forward in patient management. By tailoring the treatment to our specific healthcare context and leveraging international collaboration for training and expertise, we have successfully provided effective treatments for patients suffering from CTEPH, offering options in cases where PEA may not always be available.

The objective of this report is to present the initial data of BPA program’s implementation in Latvia and to thoroughly assess the safety and effectiveness of the first BPA sessions performed in Latvian CTEPH patients treated between 2022 and 2023. 

## 2. Materials and Methods

The current study was conducted as part of the Fundamental and Applied Research project, a state-funded research initiative with the overarching goal of fostering novel knowledge and technological advancements across various scientific domains. The primary objective of our project was to introduce and execute BPA procedures within the local medical framework, subsequently evaluating its safety and efficacy in CTEPH patients. The BPA procedures in this study were carried out by two highly experienced Latvian interventional cardiologists, each with a deep well of expertise in the field of interventional cardiology. These specialists have a cumulative experience of 26 years in interventional cardiology, consistently performing an annual average of 250 angioplasties and 150 right heart catheterizations. Their extensive background in performing complex procedures, including transcatheter aortic valve implantations (TAVIs), atrial septal defect closures, and percutaneous valve replacements, equipped them with the necessary skills and proficiency to undertake the challenging BPA procedures. To implement the BPA program in Latvia, a collaboration was established with a BPA specialist from the European Health Center Otwock in Poland. A Polish expert, who has vast expertise with BPA procedures, was invited to perform the first four procedures with the Latvian interventional cardiologists. This joint effort was instrumental in conveying the skills and information required to launch the BPA program in Latvia. The Polish specialist, with a notable track record of performing approximately 150 BPA procedures annually and having conducted around 1100 BPA procedures in total, brought significant expertise to the project. This collaborative approach enabled a successful start to BPA adoption in Latvia, establishing the framework for a full evaluation of its potential benefits for CTEPH patients.

### 2.1. Study Patients

We conducted a prospective analysis of 9 consecutive CTEPH patients who underwent BPA at Pauls Stradins Clinical University Hospital, marking the first instances of the procedure in Latvia. These patients were treated between 1 April 2022 and 1 July 2023. 

The diagnosis of CTEPH was established after radiological detection of vascular obstructions followed by right heart catheterization according to the 2022 European Society of Cardiology (ESC)/European Respiratory Society (ERS) guidelines on the diagnosis and treatment of pulmonary hypertension [2]. The criteria of right heart catheterization were: mPAP > 20 mmHg, PAWP ≤ 15 mmHg. Ventilation/perfusion (V/Q) scintigraphy was not performed in any of these patients as it was not available in Latvia at the time of diagnosis for these patients. Vascular obstructions were assessed by classic pulmonary angiography and computed tomography pulmonary angiography (CTPA). The location of the thromboembolic lesions and comorbidities were assessed by a multidisciplinary team in order to decide the operability status of all the patients. One patient had had pulmonary endarterectomy and was referred to BPA due to residual disease post-PEA, while the rest of the patients were assessed to be inoperable. 

All patients underwent a series of preprocedural examinations, including transthoracic echocardiography, World Health Organization (WHO) functional class, 6 min walk distance (6MWD), blood oxygen saturation, right heart catheterization (performed immediately before the first BPA), and blood tests. All patients underwent thorough counselling and completed a written informed consent form. After providing their consent, patients were scheduled for the procedure. The study was conducted in line with the Declaration of Helsinki. The study was independently reviewed and approved by the Ethical Committee of the Riga Stradins University (16 February 2021; approval nr 22-2/39/2021).

### 2.2. Hemodynamic Studies—Right Heart Catheterization

Hemodynamic parameters were assessed by right heart catheterization in all patients at the start of each BPA session. RHCs were performed according to recognized standards. The right atrial, right ventricular, pulmonary artery, and pulmonary capillary wedge pressures and mixed venous oxygen saturation were measured using a flexible, balloon-tipped, flow-directed catheter through a femoral vein. Measurement of cardiac output was carried out by thermodilution. Pulmonary vascular resistance, cardiac index, and stroke volume were obtained using standard formulas. 

### 2.3. Balloon Pulmonary Angioplasty

To optimize symptomatic relief and maximize hemodynamic benefits while efficiently utilizing available resources, we implemented a targeted approach that prioritized lower- or middle-lobe vessels as the primary targets for BPA. 

In preparation for the BPA procedure, patients discontinued previously used anticoagulation. Vitamin K antagonist (VKA) was discontinued five days before the scheduled date and transitioned to therapeutic doses of low-molecular-weight heparin (LMWH) until the day before admission. Direct oral anticoagulants (DOACs) were halted on the day preceding the procedure. The remaining concomitant medication was continued as previously administered. As per standard protocol, BPA procedures were performed by two interventional cardiologists. Under local anesthesia, venous access through femoral vein using the Seldinger technique was obtained. After intravenous unfractionated heparin administration, right heart catheterization was performed using a Swan–Ganz catheter followed by selective pulmonary artery catheterization. At the start of the procedure, 2000 to 2500 IU unfractionated heparin was administered, and an additional 1000 IU of heparin was given after one hour if the intervention extended beyond the anticipated timeframe. Next, the desired pulmonary arterial segment was catheterized using a 6-French 100 cm guide catheter (Medtronic Launcher JR 4.0; Minneapolis, MN, USA), and selective pulmonary angiography was performed. Iodinated contrast media was used, administered by hand injection, using a 1:2 saline dilution. Next, the target lesion was crossed using a 0.014-inch guidewire (Asahi SION Blue ES, Asahi Intec, Seto, Aichi 489-0071, Japan; Choice floppy, Boston Scientific Corporation, Marlborough, MA 01752, USA) and balloon inflations were performed using 1.5 to 4.0 mm diameter semi-compliant balloons (Emerge, Boston Scientific, Marlborough, MA, USA; Trek Abbot Vascular, Santa Clara, CA, USA). Continuous oxygen saturation monitoring was ensured during each session, and supplemental oxygen was given to maintain a saturation of 93%. 

Multiple BPA sessions were planned to minimize the use of contrast medium and reduce radiation exposure. Each consecutive BPA session was planned during the following 3–4 months. The primary goal of each balloon treatment was to achieve adequate dilation of the lesion and promptly restore pulmonary venous return. Undersized balloon catheters with a balloon/artery ratio of 0.4–0.8 were employed during the initial sessions to minimize the potential for reperfusion lung injury. 

### 2.4. Assessment of Complications and Follow-Up 

A proactive strategy for patient monitoring was used during the follow-up period to ensure the quick detection of any complications following the BPA procedure. Patients were subjected to close observation for a duration of 2 days following the BPA operation in order to detect and manage any emergent complications. Two experienced interventional cardiologists, along with an internal medicine specialist, assessed the results and possible complications following each BPA procedure. Patients were monitored to detect various complications, including but not limited to hemoptysis, infection, newly acquired arrhythmias or rhythm disturbances, pulmonary edema, allergic reactions, kidney-related issues, pulmonary vascular dissection, and pulmonary vascular perforation. To screen for complications, patients’ vital signs were monitored and they underwent physical examination every 8 h, as well as a series of blood and screening tests: complete blood count, C-reactive protein (CRP), creatinine, pulmonary angiography, chest radiography, and 12-lead electrocardiograms. The possible complications of pulmonary edema, pulmonary vascular dissection, and pulmonary vascular perforation were screened using pulmonary angiography within 15 min after the lesion dilatation. We employed blood tests for screening potential infections, with a particular focus on monitoring elevated levels of leukocytes and CRP, which would prompt a more thorough examination and diagnostic investigation to pinpoint the origin of the infection and to address the most suitable treatment. A possible infection was defined as leukocyte count >10,000/mm^3^ and CRP > 10 mg/dL. Creatinine levels were employed as a screening tool to assess kidney-related issues, and an increase in creatinine exceeding 10 percent was deemed a notable and medically significant observation, which would trigger a need for further assessment of kidney function to investigate potential kidney complications. To detect newly acquired arrhythmias or rhythm disturbances, a 12-lead ECG was employed as a screening tool. Hemoptysis and allergic reactions were evaluated by physical examination. Before the patient was discharged from hospital, a chest radiography was performed to detect lung field opacities suggestive of infiltrates, signs of pulmonary edema, or pleural effusions. Additionally, patients were monitored for the presence of various complications during the follow-up period through a combination of telemedicine visits and on-site follow-up visits at the 3-month mark. Telemedicine sessions were arranged every two weeks for each patient, allowing for ongoing examination of any signs or symptoms suggestive of potential complications. In case of any possible complications, patients would be administered to hospital for closer evaluation. Patients were invited to return for a follow-up appointment at three months after each BPA session to assess the response to treatment. Following the on-site follow-up visit at three months, no subsequent telemedicine visits were conducted during the follow-up period. At the follow-up visit, we documented and analyzed various aspects—BNP level, World Health Organization (WHO) functional class, 6 min walk distance (6MWD), and blood oxygen saturation. For two patients on whom repeated BPA sessions were performed, we additionally assessed the following hemodynamic parameters—cardiac output (CO), pulmonary vascular resistance (PVR), and mean pulmonary artery pressure (mPAP). Right heart catheterization for these patients was performed at baseline and right before each consecutive BPA session.

### 2.5. Statistical Analysis

Appropriate descriptive statistics were used to describe the study data set. Shapiro–Wilk test was used for normality tests. Paired-sample t-test was used for all normally distributed variables; otherwise, Wilcoxon signed-rank test was used. We statistically assessed the World Health Organization (WHO) functional class, 6 min walk distance (6MWD), blood oxygen saturation (SpO_2_), and BNP level at baseline and 3 months after the first BPA session. A *p*-value of less than 0.05 was deemed statistically significant. Statistical analysis was performed using IBM SPSS statistical software V.27.0 (SPSS Inc., Chicago, IL, USA). 

## 3. Results

### 3.1. Baseline Characteristics 

A total of 12 BPA procedures for nine patients were performed; repeated BPA sessions were performed for two patients. The mean age of enrolled patients was 68.8 years; 67% (*n* = 6) were female, 33% (*n* = 3) were male. Overall, 66.7% (*n* = 6) of them were in functional class III (WHO), while the rest of the patients, 33.3% (*n* = 3), were in functional class II (WHO). The median 6 MWD at baseline was 300 m. BNP levels ranged from 16 pg/mL to 864 pg/mL, with a median (IQR) of 289 (704) pg/mL. The patient baseline hemodynamic results: median mPAP 43 mmHg, PVR 7.85 WU and mean CI 2.3 L/min×m^2^ constituted advanced disease. 

Eight patients had initially inoperable disease, and one patient had recurrent PH post-PEA. All patients were receiving PAH-specific therapy as riociguat is not currently reimbursed in Latvia. All patients were receiving the phosphodiesterase-5 (PDE5) inhibitor Sildenafil, while three patients (33.3%) were on dual therapy (Sildenafil and endothelin receptor antagonist (ERA) Ambrisentan) and one patient (11.1%) was on the triple therapy of Sildenafil, Ambrisentan, and subcutaneous injections of the prostacyclin analogue Treprostinil. All patients were receiving anticoagulant therapy, where most of the patients (*n* = 5, 55.6%) received the DOAC rivaroxaban and the rest of the patients (*n* = 4, 44.4%) received the VKA warfarin. 

Almost all patients had cardiovascular comorbidities—systemic arterial hypertension and dyslipidemia being the most frequent ones, followed by coronary artery disease. The assessment of concomitant diseases and CTEPH risk factors are depicted in Table 1.

### 3.2. BPA Procedures and Outcomes 

During the course of the study, 12 BPA sessions were performed; the median number of sessions per patient was 1 (ranging from 1 to 3). One patient underwent two BPA sessions, and another undertook three BPA sessions, with 3–4 months in between each session. The median number of vessels targeted per intervention was three. Furthermore, the median procedure time was 1 h and 35 min, with a median total fluoroscopic time of 32 min (ranging from 19 to 48 min). Additionally, the median amount of iodine contrast used in these procedures was 200 mL. The efficacy of BPA on functional capacity, oxygen requirement, and BNP levels is summarized in Table 2.

**Figure 1:**
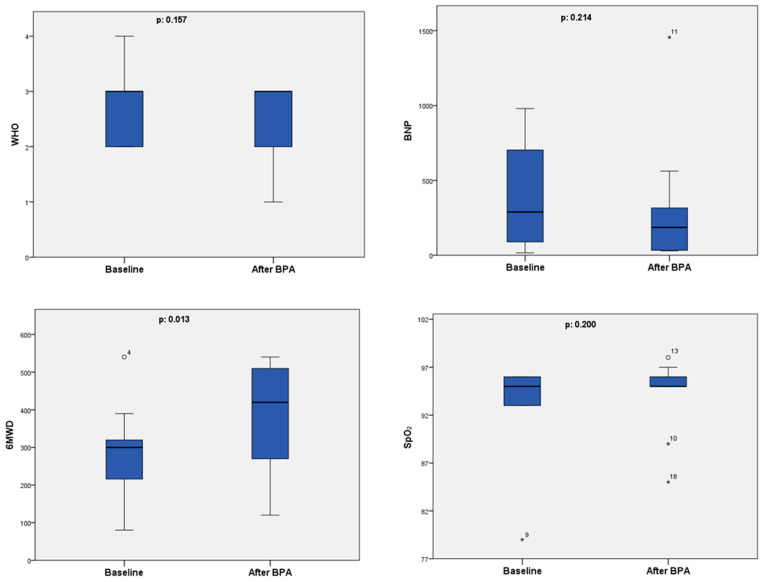
Box plots showing changes in WHO functional class, BNP, SpO_2_, and 6MWD after the first BPA session. Abbreviations: WHO—World Health Organization, BPA—balloon pulmonary angioplasty, SpO_2_—blood oxygen saturation, 6MWD—6 min walk distance, BNP—brain natriuretic peptide, ο—outlier data, *—extreme outlier data.

We assessed the statistical significance of the improvement in WHO functional class, 6MWD, BNP level, and SpO_2_ after the first BPA session (Figure 1). Out of the analyzed data, only 6MWD was normally distributed, so the paired-sample t-test was used to determine statistical significance. The remaining data were analyzed using the Wilcoxon signed-rank test.

At 3 months following the first BPA session, there was a statistically significant improvement in 6MWD, where the median distance improved from 300 to 420 m (*p* = 0.013). 

Despite the fact that we observed improvement in WHO functional class in one patient after the first BPA session, with the number of patients in functional classes I/II/III/IV changing from 0/3/6/0/ to 1/2/6/0, the improvement was not statistically significant (*p* = 0.157).

The assessment of BNP level changes revealed that, although the median values decreased after the first BPA session, the improvement was not statistically significant (*p* = 0.214). There was a slight improvement in SpO_2_ levels after the first BPA session in almost all the patients, and the mean value changed from 93% to 94%, but this change was calculated to be statistically insignificant (*p* = 0.200). As each consecutive right heart catheterization was performed right before the next planned BPA, the data for hemodynamic improvement are available from two patients—those on whom repeated BPA sessions had been performed until 1 July 2023. Due to the small sample size, we could not determine the statistical significance of the hemodynamic improvement for these patients, but we could clearly appreciate that there was a hemodynamic improvement after BPA. The mean pulmonary artery pressure (mPAP) decreased from 43 mmHg at baseline to 34.5 mmHg after the first BPA session, pulmonary vascular resistance (PVR) decreased from 7.85 WU to 4.49 WU, cardiac index (CI) increased from 2.30 ± 1.48 L/min/m^2^ to 2.85 ± 0.49 L/min/m^2^, and cardiac output (CO) increased from 5.08 L/min to 5.54 L/min, respectively. 

### 3.3. Complications

During the entire study duration, reassuringly, no fatal or severe complications necessitating tracheal intubation with mechanical ventilation occurred. Furthermore, with the exception of a single occurrence of mild hemoptysis in one patient, no other complications such as vascular damage, reperfusion edema, or other adverse effects were observed after the BPA procedures.

## 4. Discussion

We report the results of the first experience of BPA in a single center in Latvia. During the course of this study, we performed and evaluated the safety and efficacy of 12 BPA sessions for nine patients, introducing this novel technique for the treatment of CTEPH in Latvian patients. Our data support the concept that BPA is an effective interventional treatment, improving both the pulmonary hemodynamics and functional status. Moreover, we observed that BPA was safe and well tolerated. 

BPA is increasingly recognized as a viable treatment for patients suffering from chronic thromboembolic pulmonary disease, with or without associated pulmonary hypertension. The latest guidelines from the European Society of Cardiology on pulmonary hypertension now give BPA a Class 1 recommendation for cases of inoperable and persistent chronic thromboembolic pulmonary hypertension [2]. Consequently, there is a swift movement among chronic thromboembolic pulmonary hypertension centers to integrate BPA into their treatment offerings. Despite this momentum, the training for BPA is predominantly informal, reliant on knowledge exchange and hands-on training within peer-led educational initiatives, highlighting an urgent need for formalized training protocols [25]. While long-term data after BPA are currently lacking, and PEA may reduce the pulmonary pressures more than BPA, comparatively similar exercise tolerance improvements can be observed [24].

The complexity of BPA stems not only from the technical execution but also from patient selection and post-procedural care. Technical complexities lie in successful cannulation of segmental origins due to anatomical variations, usage of guide extension catheters, and correct lesion assessment during careful selective invasive pulmonary angiography and correct pressure gradient measurements for vessels appearing angiographically patent. In patient selection, experienced multidisciplinary teams are crucial for confirming diagnosis and guiding patient management, as well evaluating the clot burden, lesion classification, and the degree of hemodynamic derangement [25].

In Latvia, where PEA is unavailable, introducing BPA has been a critical advancement. This is particularly significant in our context, where patients otherwise need management abroad for PEA. Our initiative to adopt BPA, despite the challenges of the need for specialized training, significantly progresses patient management, providing a crucial treatment option within our healthcare system.

There are several studies that have looked at the safety and efficacy of BPA. Many of these report a high rate of complications, such as pulmonary edema, vessel injury/rupture, pulmonary hemorrhage, and hemorrhagic pleural effusion [26,27,28,29,30]. Even in expert centers in Japan, BPA-related complications occur with a frequency of up to 12% [26]. In a Japanese multicenter registry, where data were presented from 308 patients and 1408 procedures from seven centers, BPA-related complications occurred in 36.3% patients and 30-day mortality was 2.6%, the main causes of death being right heart failure, multiorgan failure, and sepsis [27]. Data from San Diego Hospital, USA, where BPA efficacy and safety were assessed over a 6-year period for 643 BPA sessions for 153 patients, showed no BPA-related mortalities, and BPA-related complications were observed in 9.2% patients [28]. A prospective, multicenter registry of adult and pediatric PAH and CTEPH in Poland analyzed data from 263 patients, where over the 5-year period 1056 BPA procedures were performed [29]. The data showed that 3.3% of patients had vascular injury, 3.0% had wire perforation, and 1.5% vascular dissection during the BPA procedure and 6.4% of patients had lung injury, 2.8% had renal dysfunction, and 1.3% of patients had infection after the BPA procedure [29]. Overall, BPA-related complications vary from center to center and are more likely to be reported when a higher volume of BPA sessions is performed. Our study showed an excellent initial safety profile, with no observed complications (except for one patient with hemoptysis) after or during the first BPA sessions, although more BPA procedures must consecutively be performed to assess this appropriately. 

The baseline characteristics of the chosen cohort of CTEPH patients for BPA procedures exhibit consistency with the overall baseline characteristics observed amongst CTEPH patients in Latvia across time. Notably, the mean age of the selected BPA recipients was at 68.8 ± 11.8 years, a distribution that reflects the typical age range of the overall Latvian CTEPH patient cohort, which spans from 67.0 to 72.8 years [4,31,32]. Correspondingly, the BNP levels in BPA recipients (289.00 ± 379.26 pg/mL) mirrors the baseline characteristics seen in Latvian CTEPH patients (340.00–607.70 pg/mL) [31,32,33]. Moreover, the 6MWD among BPA recipients was 300.00 ± 133.47 m, which aligns with the general distribution observed in Latvian CTEPH patients, ranging from 244 to 300 m [4,31,32]. This consistency underlines the cohort’s fidelity in representing the broader patient demographic. A notable alignment was also observed in hemodynamic parameters. The selected BPA recipients demonstrate values for mPAP 43 ± 15.56 mmHg and PVR at 7.85 ± 2.89 Wood units (WU), mirroring the baseline hemodynamic characteristics of CTEPH patients in Latvia, where mPAP ranges between 40 and 51 mmHg and PVR ranges from 7.35 to 10.30 WU [4,31,32]. However, the CO (5.08 ± 0.37 L/min) and CI (2.30 ± 1.48 L/min/m^2^) parameters in the selected BPA recipients were greater than those of the overall CTEPH population in Latvia, where CO ranges from 3.7 to 4.7 L/min and CI ranges from 1.9 to 2.5 L/min/m^2^ [4,31,32]. The observed similarities in baseline characteristics between BPA recipients and the overall CTEPH population in Latvia increases this study’s ability to offer comprehensive insights into the BPA procedure for CTEPH patients within the Latvian healthcare system.

The median procedure duration was 1 h and 35 min. Many experienced centers show longer procedure times; for example, a Portuguese PH expert center [34] reported a mean procedure time of 124 ± 30 min, data from a Spanish BPA program show a mean procedure time of 123 ± 25 min [35]. Many of these centers additionally perform intravascular imaging during sessions that was not used in our center. The median volume of iodine contrast medium used in our center was 200 mL, and median fluoroscopy time was 32 min per BPA session. Japanese colleagues [22] report that they require 160.2 ± 57.2 mL of contrast medium per session and recommend that it should not exceed 300 ml per session [36]. Even though our center shows higher contrast material usage, it does not exceed the recommended level. Our total fluoroscopy time also does not exceed the recommended level and is in line with the clinical consensus of the BPA methodology standard that has been recently published by the European Society of Cardiology working group on pulmonary circulation and right ventricular function [37].

The average (median) 6MWD improvement in our study was +120 m (40% increase from baseline), and WHO functional class improvement was observed in one patient, corresponding to 11% of patients. It is important to add that WHO functional class did not deteriorate in any of our patients.

A publication from Germany, where the initial BPA experience was outlined, showed a mean 6MWD improvement of +33 m (9% from baseline) and WHO functional class improvement in 59% of patients, although measurements were performed after an average of five BPA sessions [38]. In a recent publication evaluating the efficacy and safety of BPA, an analysis of BPA treatment outcomes in two collaborating CTEPH referral centers in Poland, characterized by similar geographical and baseline clinical characteristics to Latvian CTEPH patients, showed improvement in mean 6MWD of 68 m and change in WHO FC from (I–II 20%/III–IV 80%) to (I–II 77%/III–IV 23%) [39]. Other studies show improvements of 6MWD on average of 30–70 m, and a WHO functional class increase in most patients [22,27,40,41,42,43]. Our results show an improvement in WHO functional class and a significant 6MWD improvement after the first BPA session; these results are promising, particularly because both attributes contribute to increasing quality of life. It is thought that exercise capacity, alongside hemodynamics and heart failure symptoms, improves with the number of procedures performed (and number of treated vessels) [44]. So, we expect that after the final BPA session for each patient, these results will have improved even more.

Despite the fact that there was no statistically significant improvement in BNP, WHO functional class, and blood oxygen saturation after BPA, a strong positive trend was observed. It has been shown that BNP/NT-pro-BNP correlate with myocardial stress and are predictors of disease severity and prognosis of pulmonary hypertension [2,45,46]. As with our results, we observed a reduction in BNP levels of 103 pg/mL (35.6% from baseline), demonstrating a reduction in right ventricular wall stress after BPA. Other centers have shown improvements in mean NT-pro-BNP from 220 to 1101 pg/mL [2,39,42] and mean BNP from 60.8 to 196.2 pg/mL after the BPA procedures [27,40,43].

BPA also resulted in an increase in blood oxygen saturation measured in room air, from an average 93% at baseline to 94% after BPA. No oxygen supplementation was needed for any of our patients, neither prior nor after BPA. 

As discussed above, no adverse events occurred in any of our patients, and mortality rate was 0%. One of the most common complications after BPA is pulmonary bleeding; moreover, German colleagues used LMWH bridging in patients receiving DOACs [39]. This was not performed in our center and might have reduced the potential of pulmonary bleeding. Our findings indicate a respectable initial result for a single center but would have to be reassessed when a more comparable number of patients are treated. 

Several limitations should be acknowledged in the interpretation of our study’s results. While the number of enrolled patients in our study is limited and poses challenges to the broader generalizability of our findings, it is crucial to emphasize that the limited cohort size is attributed to the financial constraints of the grant funding, which facilitated only 12 BPA procedures. Notably, the current absence of reimbursement for the BPA procedure within the Latvian healthcare framework underscores the significance of this study, as efforts are underway to advocate for its inclusion as a reimbursed intervention for CTEPH patients, thus potentially expanding access and allowing for more comprehensive research in the future. Our report did not include a control group, which poses a limitation on our ability to directly compare the outcomes of BPA procedures with alternative treatments or non-interventional approaches. While our study focused on evaluating the initial experience of the BPA procedure for CTEPH patients in Latvia, future research designs incorporating a control group could provide a more comprehensive understanding of the procedure’s relative effectiveness in Latvia. Another important limitation to note is that none of the patients included in our study completed the entire planned series of BPA sessions. As a result, the comprehensive assessment of BPA’s long-term efficacy and safety within the context of the Latvian CTEPH population remains a work in progress. The incomplete data on the full treatment course introduce a temporal limitation to our study’s scope, emphasizing the need for further investigation and follow-up to provide a more robust evaluation of the procedure’s outcomes.

## 5. Conclusions

Our report outlines the first experience of BPA in a single center in Latvia, introducing this novel technique for the treatment of CTEPH in Latvian patients. Our data reveal that BPA could be an effective and safe interventional treatment, improving the functional status of CTEPH patients. These findings show positive initial results for a single center, but more patients need to be treated in order to assess the long-term effects and safety of BPA. 

## Figures and Tables

**Table 1:** Prevalence of comorbidities and CTEPH-specific risk factors in the study cohort. Each condition is listed alongside the number and percentage of patients affected.

Concomitant Diseases	Number of Patients, (%)
Systemic arterial hypertension	7 (77%)
Dyslipidemia	7 (77%)
Diabetes	3 (33%)
Coronary artery disease	4 (44%)
COPD	0
Chronic kidney disease	1 (11%)
Atrial fibrillation	1 (11%)
CTEPH risk factors	
History of acute pulmonary embolism	9 (100%)
History of deep vein thrombosis	2 (22%)
Recurrence of pulmonary embolism	1 (11%)
Chronic inflammatory disease	0
Atrial septal defect	0
VA shunt	0
History of splenectomy	0
Antiphospholipid syndrome	0
History of malignancy	0

Abbreviations: COPD—chronic obstructive pulmonary disease, CTEPH—chronic thromboembolic pulmonary hypertension, VA—ventriculoatrial.

**Table 2:** Characteristics at baseline and 3 months after the first BPA session.

Total no. patients	9	
Age (years)	68.8 ± 11.8	
Female sex (%)	67	
Total no. procedures	12	
Median no. procedures per person	1	
	**Baseline**	**After BPA**
6MWD (m)	300.00 ± 133.47	420.00 ± 147.23
FC (WHO) I/II/III/IV	0/3/6/0	1/2/6/0
BNP (pg/mL)	289 (704)	186 (407)
SpO_2_ (%)	93.11 ± 5.41	94 ± 4.21
30-day mortality (%)	-	0
**Hemodynamic characteristics**		
mPAP (mmHg)	43.00 ± 15.56	
CO (L/min)	5.08 ± 0.37	
CI (L/min/m^2^)	2.30 ± 1.48	
PVR (WU)	7.85 ± 2.89	

Abbreviations: BPA—balloon pulmonary angioplasty, 6MWD—6 min walk distance, mPAP—mean pulmonary artery pressure, CO—cardiac output, CI—cardiac index, PVR—pulmonary vascular resistance, WHO—World Health Organization, FC—functional class, BNP—brain natriuretic peptide, SpO_2_—blood oxygen saturation.

## Data Availability

The datasets used and analyzed for this study are available from the corresponding author upon a reasonable request.
